# A Denoising Algorithm for Maglev Gyro Jump Data Based on Bayesian Ensemble Time-Series Segmentation

**DOI:** 10.3390/s26165287

**Published:** 2026-08-20

**Authors:** Binqiang Guo, Zhen Shi, Di Liu, Xinkang Hu, Gang Jiang, Tao Dang

**Affiliations:** 1 Shaanxi Huashan Road & Bridge Group Co., Ltd., Xi’an 710016, China; shangboming2000@163.com (B.G.); sx952019@163.com (X.H.); taizi702@163.com (T.D.); 2 School of Land Engineering, Chang’an University, 126 Yanta Road, Xi’an 710054, China; di.liu@chd.edu.cn (D.L.); chdjiang@chd.edu.cn (G.J.); 3 Shaanxi Zhengcheng Road & Bridge Engineering Research Institute Co., Ltd., Xi’an 710086, China

**Keywords:** maglev gyroscope, north-seeking, BEAST, optimized wavelet transform, jump signal denoising

## Abstract

High-precision tunnel breakthroughs depend critically on the north-seeking accuracy of maglev gyroscopes. However, external disturbances during underground construction often introduce abrupt jumps into rotor current signals, significantly reducing the orientation reliability. Existing signal-processing methods either require manually defined segmentation windows or apply identical denoising strategies to both stationary and disturbed signal intervals, resulting in limited adaptability and suboptimal denoising performance. To overcome these limitations, this study proposes an improved rotor current denoising algorithm based on the MAF-ARIMA framework by incorporating the Bayesian ensemble algorithm for abrupt change, seasonality, and trend (BEAST) and an optimized wavelet transform (OWT). First, the BEAST is employed to automatically detect the structural change point of the rotor current signal, enabling the adaptive segmentation of stationary and jump intervals without manual intervention. Subsequently, empirical mode decomposition is performed, and the OWT applies different denoising parameters to the dominant components of the stationary and jump segments according to their distinct fluctuation characteristics. Finally, moving-average smoothing is adopted to preserve the signal continuity at the segmentation boundary, while the autoregressive integrated moving average (ARIMA) model reconstructs the missing trend component of the jump interval to obtain the complete denoised signal. Comparative experiments using 12 field-collected rotor current datasets demonstrated that the proposed method reduced the standard deviation of the denoised signal by 70.96% and the absolute azimuth error by 50.36% compared with the raw signal, outperforming the optimized Hilbert–Huang transform, HSA-KS, and the original MAF-ARIMA algorithm. By introducing adaptive change-point detection and segment-specific denoising into the existing MAF-ARIMA framework, the proposed method significantly improves the adaptability and denoising performance of maglev gyro rotor current processing under complex tunnel construction environments while preserving the signal continuity and reconstruction accuracy.

## 1. Introduction

High-precision penetration is crucial in the construction of underground engineering projects such as railway tunnels, hydraulic tunnels, and mine tunnels [1,2]. The complex internal environment and limited construction conditions of tunnels lead to the gradual accumulation of underground azimuth measurement errors with tunnel excavation, which not only increases the risk of a penetration deviation but may also cause serious economic losses and safety accidents [3].

A gyro total station is a high-precision inertial measurement instrument that can independently determine the geographic azimuth by inducing the pointing torque generated by the Earth’s rotation on the gyro rotation axis, providing a reliable azimuth reference for tunnel penetration [4]. Traditional suspended-band gyro total stations achieve north-seeking by dynamically tracking the meridian [5], but they can only provide a single direction measurement for the directional side, which limits the reliability and accuracy of directional achievements to a considerable extent. In contrast, the maglev gyro total station, based on the principle of torque balance, measures the north azimuth angle of any survey line by measuring the reverse torque [6]. This instrument can obtain large amounts of observation data for analyses and processing, enabling the accuracy and reliability of gyro orientation to be improved. Therefore, it has been widely used in many major tunnel engineering projects, such as the Hong Kong–Zhuhai–Macao Bridge [7,8].

The construction environment of underground tunnels is complex, and external disturbances such as on-site airflow disturbances and equipment vibration often break the torque balance state of the gyro, causing the interference noise caused by external vibration to mix into the north-seeking data collected by the torque sensor. This makes it difficult to ensure the gyro orientation accuracy. Therefore, denoising processing of gyro sampling signals is particularly important. Relevant studies have shown that the rotor current signal in maglev gyro north-seeking data is extremely sensitive to changes in the external environment, and external instantaneous disturbances result in the most significant interference to maglev gyro north-seeking data. The rotor current signal will have abnormal jumps under the influence of external instantaneous vibration, which greatly reduces the reliability of north azimuth angle measurement results [9,10].

In recent years, relevant scholars have conducted in-depth research on rotor current signal-processing methods under the interference of external instantaneous disturbances. Ma Ji determined the optimal reconstruction order (IMF order of eight) of empirical mode decomposition based on the marginal spectrum energy weighting algorithm, and proposed an improved Hilbert–Huang transform rotor current signal-filtering algorithm (OHHT) [11]. This algorithm effectively reduces the north azimuth angle measurement error, but due to the high order of signal decomposition and reconstruction, there is a risk of reconstructed signal distortion, and the adaptive ability of the algorithm is weak. Wang Yiwen proposed a new method (HSA-KS) combining a heuristic segmentation algorithm (HSA) and a two-sample Kolmogorov–Smirnov (KS) test, which enables the correction of gyro signals under the influence of instantaneous external disturbances by adaptively detecting and eliminating jumps [12]. However, eliminating abnormal jump data reduces the amount of initial data for north azimuth angle measurements and destroys the continuity of the rotor current signal. Therefore, the effectiveness of the HSA-KS method in processing rotor current signals that contain abnormal jumps needs to be further verified. Liu Di proposed a rotor current signal-processing method (MAF-ARIMA) that combines moving average filtering (MAF) and an autoregressive integrated moving average model (ARIMA) [13]. This method effectively reduces the influence of external instantaneous disturbances on maglev gyro north-seeking results by predicting the missing trend items corresponding to abnormal jump interval data; experimental field results verified the effectiveness of the method. Although the MAF-ARIMA method can ensure the integrity of north azimuth angle measurement data and the continuity of rotor current signals, it requires the manual division of fixed window sizes during data-sampling-interval property judgment, resulting in low adaptability. In addition, the same filtering method with the same parameter settings is adopted for stationary data and jump data, ignoring the difference in the degree of external interference between stationary data and jump data. Therefore, the MAF-ARIMA algorithm still has certain limitations.

Existing studies have made significant progress in suppressing abnormal jumps in maglev gyro rotor current signals; however, three critical limitations remain unresolved. First, the current approaches generally rely on manually determined window lengths or fixed statistical criteria for signal segmentation, which limits their adaptability when disturbance characteristics vary under different construction environments. Second, most existing denoising methods either directly remove abnormal jump samples or process the entire signal using identical filtering parameters, resulting in discontinuities in the reconstructed signal or the insufficient preservation of local signal characteristics. Third, although prediction-based approaches improve the signal completeness, they still employ fixed-parameter filtering strategies for different signal states, making them less effective when the disturbance intensity changes dynamically. These limitations motivate the development of a more adaptive and segmented denoising framework.

To address the above limitations, this study proposes an improved MAF-ARIMA framework incorporating the Bayesian ensemble algorithm for abrupt change, seasonality, and trend (BEAST) and an optimized wavelet transform (OWT). Unlike previous methods that employed fixed segmentation criteria, the proposed method first performs automatic time-series segmentation using probabilistic change-point detection to distinguish stationary and jump intervals. Subsequently, empirical mode decomposition (EMD) is applied separately to each segment so that dominant signal components can be extracted according to their local characteristics. Adaptive wavelet filtering with differentiated parameter settings is then adopted to suppress noise while preserving the signal continuity through moving-average smoothing. Finally, missing trend information in the jump intervals is reconstructed using an ARIMA prediction model trained on stationary data. By integrating adaptive segmentation, differentiated filtering, signal continuity enhancement, and trend reconstruction into a unified framework, the proposed method improves the adaptability to varying disturbance conditions while overcoming the limitations associated with fixed-parameter denoising strategies.

The main contributions of this study are summarized as follows:

(1) A BEAST-based probabilistic time-series segmentation strategy is introduced to automatically identify rotor current change points without requiring manually defined segmentation windows, thereby improving the adaptability under varying disturbance conditions.
(2) A segmented adaptive denoising framework is proposed in which stationary and jump intervals are processed independently using EMD-based dominant component extraction and optimized wavelet filtering with different parameter settings. This strategy effectively suppresses noise while preserving the signal continuity.
(3) Comprehensive experiments conducted on 12 groups of field-collected rotor current signals demonstrated that the proposed method achieved a superior denoising performance compared with the OHHT, HSA-KS, and MAF-ARIMA in terms of both the internal and external compliance accuracy, while further improving the adaptability and robustness.

Compared with the previously proposed MAF-ARIMA method, the proposed method introduces three major methodological improvements. First, signal segmentation is automatically achieved using Bayesian probabilistic change-point detection instead of manually predefined statistical windows. Second, the stationary and jump intervals are processed using differentiated adaptive filtering strategies rather than identical filtering parameters, thereby improving the local denoising performance. Third, the signal continuity is explicitly preserved through breakpoint smoothing before trend reconstruction, whereas previous methods mainly focused on trend prediction. These improvements enable the proposed method to better accommodate complex disturbance patterns encountered during practical tunnel construction.

The remainder of this paper is organized as follows. Section 2 presents the proposed methodology in detail, including BEAST-based time-series segmentation (Section 2.1), segment-wise signal decomposition (Section 2.2), dominant-component identification and adaptive filtering (Section 2.3), breakpoint smoothing and continuity preservation (Section 2.4), trend prediction and signal reconstruction (Section 2.5), evaluation metrics (Section 2.6), and the complete implementation procedure (Section 2.7). Section 3 describes the field experimental setup and data acquisition (Section 3.1), reports representative signal processing results (Section 3.2 and Section 3.3), presents comparative performance on all test signals with statistical significance tests (Section 3.4), and provides ablation study results to quantify the contribution of each module (Section 3.5). Section 4 concludes the paper with a summary of findings and discusses limitations and directions for future work.

## 2. Proposed Methodology

### 2.1. BEAST-Based Time-Series Segmentation

Time-series segmentation is the core decision stage of the proposed method. Its purpose is not merely to detect isolated outliers, but to identify the temporal boundary at which the statistical trend of the rotor current changes because of an external instantaneous disturbance. The detected boundary divides the original sequence into intervals with different signal states, enabling different decomposition and filtering strategies to be assigned to the stationary and jump data.

This strategy differs from sample-level jump elimination. Individual samples after the boundary are not directly classified as invalid or removed. Instead, all samples are retained and assigned to a segment according to the posterior evidence of a structural trend change. Therefore, this method preserves the original time index, sampling length, and azimuth-related information contained in the disturbed interval.

Compared with conventional change-point detection approaches such as CUSUM, binary segmentation, and PELT, the BEAST estimates the posterior probability of structural changes under a Bayesian ensemble framework rather than relying on fixed statistical thresholds. This probabilistic formulation enables uncertainty quantification and allows change points to be identified according to the overall trend evolution instead of isolated local fluctuations. Since rotor current jumps induced by external disturbances often exhibit different amplitudes and durations under practical tunnel environments, the probabilistic segmentation strategy of the BEAST provides greater adaptability than threshold-based methods whose detection performance is highly dependent on manually selected parameters.

The BEAST is a change-point detection algorithm based on Bayesian ensemble theory, specifically designed to identify structural mutation points in time series [14]. By aiming at the jump characteristics of rotor current data in the maglev gyro north-seeking system, this algorithm provides complete statistical inference of the position, quantity, and uncertainty of jump points by integrating multiple potential change-point configurations [15].

The BEAST models the rotor current time series of the maglev gyro as a piecewise stationary process:

(1) $$y_{t}=f(t)+\varepsilon_{t},\varepsilon_{t}∼N(0,\sigma^{2})$$

where $y_{t}$ represents the rotor current value at the *t*-th sampling point and f(t) is the piecewise trend function with structural changes at jump points. Based on the Bayesian model-averaging framework, the algorithm calculates the posterior probability of all possible change-point configurations:

(2) $$P(\tau |y)=\sum_{M\in \Omega }P(\tau |M,y)\cdot P(M|y)$$

where Ω represents the space of all possible change-point configurations and P(M|y) is the posterior probability of the model.

The posterior distribution is estimated by decomposing the observed sequence into trend, seasonal, and residual components.

By examining the data characteristics of the maglev gyro rotor current, the BEAST decomposes the rotor current time series into the following:

(3) $$y_{t}=T_{t}+S_{t}+\varepsilon_{t}$$

where $T_{t}$ is the trend component, modeled as a piecewise linear function to capture the long-term change in the rotor current; $S_{t}$ is the seasonal component (the influence of periodic interference was negligible compared with the jump data in this study); and $\varepsilon_{t}$ is the observation noise, which obeys a zero-mean normal distribution.

After the trend component model processing shown in Equation (3), the prior setting and a marginal likelihood calculation are carried out. In the prior distribution setting, a truncated Poisson prior is adopted to determine the number of change points; based on the uniform time distribution, it is assumed that change points are equally likely to occur at each sampling point. A normal-gamma conjugate prior is set for piecewise slopes and intercepts to determine the trend parameters. For a given change point configuration, the marginal likelihood function is as follows:

(4) P(y|M)=∫P(y|θ,M)P(θ,M)dθ

where y is the observation data vector, representing the rotor current sequence of the maglev gyro; θ is the model parameter set, which includes the piecewise intercept parameters (reflecting the baseline level of rotor current in the segment), the piecewise slope parameters (describing the change trend of the rotor current in the segment), and the noise variance (characterizing the observation error and system noise) in the context of a maglev gyro application.

The reversible-jump MCMC method is used to explore the change-point space. By collecting *N* posterior samples, the posterior probability of each position as a jump point is calculated:

(5) $$p_{t}=\frac{1}{N}\sum_{i=1}^{N}\prod \left(t\in \tau^{\left(i\right)}\right)$$

where N is the total number of MCMC posterior samples; $\tau^{\left(i\right)}$ is the change point set in the *i*-th sample; and ∏(⋅) is the indicator function.

After MCMC sampling, a posterior change-point probability sequence is obtained for all sampling positions. Let $p_{t}$ denote the posterior probability that the *t*-th position is a structural change point. The candidate positions are first identified from local probability peaks. The segmentation boundary is then determined by jointly considering three conditions:

(1) The posterior change-point probability exhibits a distinct local peak;
(2) The fitted trend before and after the candidate position shows an evident level or slope change;
(3) The trend variation persists over the subsequent sampling interval rather than appearing as an isolated transient fluctuation.

The first candidate to satisfy these conditions is selected as the boundary $t_{b}$. The original rotor current sequence x(t) is consequently divided as

(6) $$\begin{array}{l} X_{s}=\left\{x(t)\left|1\leq t\leq t_{b}\right.\right\}\\ X_{j}=\left\{x(t)\left|t_{b}\leq t\leq N\right.\right\} \end{array}$$

where $X_{s}$ and $X_{j}$ denote the stationary and jump intervals, respectively, and N is the total number of samples.

The boundary is therefore obtained from posterior structural evidence rather than from a preset window size. Once $t_{b}$ is determined, all subsequent EMD, component-screening, wavelet-filtering, and trend-processing operations are independently performed for $X_{s}$ and $X_{j}$. This establishes an explicit connection between Bayesian segmentation and differentiated denoising.

The rationale for selecting the first persistent trend-change candidate as the segmentation boundary, rather than subsequent candidates, is grounded in both the physical mechanism of gyro operation and the empirical characteristics of field disturbance signals. When an instantaneous external disturbance (e.g., vibration or airflow) impacts the maglev gyro, the rotor current experiences an abrupt structural break at the very onset of the perturbation. Subsequent fluctuations often correspond to transient oscillations or recovery processes rather than additional independent disturbance events. Selecting the first statistically supported boundary ensures that the entire disturbed interval is assigned to the jump segment for differentiated processing, while any later local probability peaks within the already-disturbed region are treated as internal variations in the jump segment rather than as new segmentation boundaries. This strategy prevents over-segmentation and maintains a clean binary partition (stationary vs. jump), which is essential for the subsequent segment-wise EMD and filtering operations. Moreover, the BEAST inherently provides posterior probability estimates for all potential change points; the proposed method deliberately adopts the first persistent candidate to capture the primary disturbance onset, thereby prioritizing the integrity of the stationary reference interval over the finer-grained subdivision of the disturbed period.

Following an instantaneous disturbance, the restoring torque of the maglev gyro continuously compensates for the disturbance torque until a new torque equilibrium is gradually established. Consequently, the resulting trend variation persists over a finite recovery interval rather than appearing as an isolated transient fluctuation. Based on this physical mechanism, a candidate change point is regarded as persistent if the posterior change-point probability exceeds 0.05, and the corresponding trend does not return to its pre-change level within the subsequent observation window. In this study, the persistence criterion is evaluated over the following 1000 samples. If the posterior probability remains above 0.05 for at least one consecutive interval within this window while the estimated trend continues to deviate from the pre-change state, the candidate is considered to satisfy the persistence criterion and is eligible to be selected as the segmentation boundary. The selected window length exceeds the typical recovery duration of the rotor current following an instantaneous disturbance, thereby reducing the likelihood that transient fluctuations are incorrectly identified as structural trend changes.

### 2.2. Segment-Wise Signal Decomposition

After the segmentation boundary is determined, the stationary interval and the jump interval are decomposed independently. Separate decomposition is necessary because the two intervals exhibit different amplitudes, fluctuation intensities, and frequency characteristics. Applying EMD to the complete sequence as a single signal may cause the locally disturbed interval to affect the decomposition modes of the stationary interval. The stationary interval mainly contains measurement noise, whereas the jump interval contains both measurement noise and disturbance-induced structural variations. Therefore, identical filtering parameters are unsuitable for both intervals.

Empirical mode decomposition (EMD) is an adaptive and efficient non-stationary nonlinear signal-processing method [16]. This algorithm can decompose complex signals into a finite number of intrinsic mode functions (IMF), each representing the oscillation mode of the signal on different time scales.

Each segment $x_{k}(t)$ processed by the EMD algorithm, where $k\in \left\{s,j\right\}$, can be expressed as the sum of the IMF components and residuals [17]:

(7) $$x_{k}(t)=\sum_{i=1}^{n_{k}}IMF_{k,i}(t)+r_{k}(t)$$

where $n_{k}$ is the number of decomposed IMFs from the segment k, and $r_{k}(t)$ is the final residual term, which can be used as the trend component of the rotor current.

The decomposition is performed separately for the two segments without resampling or changing their temporal order. Thus, the sum of the segment lengths remains equal to the length of the original rotor current sequence.

### 2.3. Dominant-Component Identification and Adaptive Filtering

The Hausdorff distance is a mathematical method of measuring the similarity between two point sets; it is widely used in image processing, pattern recognition, shape matching, and other fields [18,19].

For two non-empty point sets $A=\left\{a1,a2,\ldots am\right\}$ and $B=\left\{b1,b2,\ldots bm\right\}$, the Hausdorff distance is defined as follows:

(8) H(A,B)=max(h(A,B),h(B,A))

where $h(A,B)=\max_{a\in A}\min_{b\in B}\left\|a-b\right\|$ represents the directed Hausdorff distance from *A* to *B*; $h(B,A)=\max_{b\in B}\min_{a\in A}\left\|a-b\right\|$ represents the directed Hausdorff distance from *B* to *A*; and $\left\|\;\cdot \;\right\|$ represents the distance metric (Euclidean distance was adopted in this study).

In addition, the probability density function (pdf) of the intrinsic mode function can be estimated using the kernel density [20]. The similarity between the input signal and each mode can be expressed in the form of a pdf as follows:

(9) $$L(i)=H(pdf(x(t),pdf(IMF_{i}(t))))$$

where L(i) is the similarity metric. The point next to the first maximum value in the sequence is the separation point between the noise component and the signal component, so as to realize the extraction of the signal-dominant component in the rotor current data.

For each segment, the probability density function of the original signal is compared with that of every IMF component. The Hausdorff-distance sequence reflects the similarity between the statistical distribution of an IMF and that of the corresponding segment. The point adjacent to the first pronounced maximum is used as the separation position between high-frequency noise-dominant components and signal-dominant components.

The Hausdorff distance was adopted as the criterion for distinguishing noise-dominant from signal-dominant IMF components for three reasons. First, unlike the variance contribution ratio or correlation coefficient, which primarily capture second-order statistical similarity or linear associations, the Hausdorff distance measures the maximum mismatch between two point sets, and thus reflects the global shape difference between the probability density functions of an IMF and the original signal. This property is particularly advantageous for non-stationary signals containing abrupt jumps, where the distributional shape of the signal-dominant component differs fundamentally from that of the noise-dominant component. Second, the Hausdorff distance is robust to localized probability-density fluctuations caused by additive noise, reducing the risk of misclassification due to isolated outliers. Third, the HD-based IMF screening strategy has been successfully applied in gyroscope signal denoising and other time–frequency decomposition tasks, demonstrating its effectiveness in adaptively separating informative components from noise. For these reasons, HD provides a more suitable criterion for the present application than conventional alternatives.

Because this separation is conducted independently for the stationary and jump intervals, the number of removed high-frequency IMFs is not required to be identical. This segment-dependent component selection avoids the fixed reconstruction order used by methods that process the complete signal uniformly.

After extracting the signal-dominant component from the rotor current data, the adaptive wavelet denoising algorithm is used to process this component and obtain the filtering results. The wavelet denoising algorithm constructs basis functions of orthogonal transformation using window functions. By selecting an appropriate wavelet function, the signal is decomposed into a series of high-frequency and low-frequency wavelet coefficients; then, the high-frequency coefficients are thresholded according to the corresponding threshold criterion and reconstructed with the low-frequency coefficients, so as to achieve denoising and signal correction. The Mallat algorithm is a classical decomposition algorithm of a wavelet transform, and is used to decompose signals with orthogonal scale functions and wavelet functions under two-scale coefficients. The scale function constitutes a low-pass filter, and the wavelet function constitutes a high-pass filter. The original signal is decomposed into low-frequency coefficients and high-frequency coefficients, and the low-frequency coefficients are decomposed layer by layer. Finally, the signal is decomposed into a remaining low-frequency coefficient and a series of high-frequency coefficients [21,22]. The Mallat algorithm’s formula can be expressed as follows:

(10) $$\begin{array}{l} a_{j,k}=<f,\varphi_{j,k}>=\sum_{n\in Z}f(t)\varphi_{j,k}(t),k\in Z\\ d_{j,k}=<f,\psi_{j,k}>=\sum_{n\in Z}f(t)\psi_{j,k}(t),k\in Z \end{array}$$

where *f* is the original signal; $a_{j,k}$ and $d_{j,k}$ are the decomposed low-frequency and high-frequency components, respectively; and $\varphi_{j,k}$ and $\psi_{j,k}$ are the scale function and wavelet function, respectively.

In the process of adaptive wavelet denoising, specific parameters can be determined through empirical research on the optimal decomposition order of wavelet filtering for different signal fluctuation forms [23]. The retained IMF components of each interval are superimposed to form the local signal-dominant component. Optimized wavelet filtering is then applied separately to the stationary- and jump-dominant components. The wavelet basis, decomposition level, and threshold-related settings are selected according to the fluctuation characteristics of the corresponding segment rather than being imposed uniformly on the full sequence. The objective of this differentiated processing is to avoid two opposite effects: excessive smoothing of the stationary interval and insufficient suppression of the strongly disturbed jump interval. In this way, the BEAST segmentation result directly controls the assignment of denoising parameters.

It is acknowledged that EMD suffers from the inherent mode-mixing limitation when processing signals that contain noise and abrupt jumps. In the proposed method, however, the influence of mode mixing is mitigated by two factors. First, the BEAST-based pre-segmentation ensures that EMD is applied separately to the stationary and jump intervals, each of which exhibits relatively homogeneous statistical characteristics. This reduces the likelihood that oscillations of different scales will be interleaved within the same decomposition interval. Second, the Hausdorff-distance-based component screening operates on the probability density function similarity between each IMF and the original segment, rather than relying on a fixed reconstruction order. This distribution-based criterion is less sensitive to the boundary ambiguity caused by moderate mode mixing. For the specific task of rotor current reconstruction and azimuth calculation, the residual effect of mode mixing is further suppressed by the subsequent optimized wavelet filtering and ARIMA-based trend prediction. Therefore, the use of standard EMD does not compromise the effectiveness of the proposed method.

### 2.4. Breakpoint Smoothing and Continuity Preservation

After separate filtering, the two processed segments are concatenated according to their original sampling positions. Because the filtering parameters and local statistical characteristics differ between the two segments, direct concatenation may produce an artificial discontinuity near the segmentation boundary. Such a breakpoint is introduced by segmented processing rather than by the physical rotor current signal, and should therefore be corrected.

For a time series $\left\{x_{1},x_{2},\ldots x_{N}\right\}$ of length *N* with window size m, the moving average is defined as follows [24]:

(11) $$MA_{t}=\frac{1}{m}\sum_{i=0}^{m-1}x_{t-i}$$

where $MA_{t}$ is the moving average value at the t time point and $x_{t-i}$ is the original observation value at the t−i time.

According to the orientation mechanism of the magnetically suspended gyroscope, azimuth angle calculation relies on the mean value of rotor current. The smoothing process cannot change the magnitude of the mean rotor current for large-scale samples; this method mitigates abrupt discrepancies between adjacent segments.

Unlike HSA-KS, which eliminates detected jump samples, the proposed continuity-preservation step does not shorten the sequence. Unlike MAF-ARIMA, moving-average processing is not used as a uniform denoising operator for all data; it is specifically used to correct the junction introduced by differentiated segment-wise filtering.

### 2.5. Trend-Component Prediction and Signal Reconstruction

The autoregressive integrated moving average (ARIMA) model is a classical time-series prediction method. This model can effectively deal with the prediction of non-stationary time series by combining three components: autoregression, differencing, and the moving average [25,26]. Deep-learning prediction models such as LSTM generally require substantially larger training datasets and extensive hyperparameter optimization to achieve stable performance. In contrast, the trend-prediction task considered in this study involves relatively short stationary sequences acquired under field conditions. Therefore, the ARIMA provides a computationally efficient and statistically interpretable solution that is better suited to the available data scale. Future studies may investigate whether advanced nonlinear predictors can further improve the reconstruction performance under larger datasets.

The general form of the ARIMA model is as follows:

(12) $$(1-\phi_{1}B-\cdots -\phi_{p}B^{p}){\left(1-B\right)}^{d}y_{t}=c+(1+\theta_{1}B+\cdots +\theta_{q}B^{q})\varepsilon_{t}$$

where $y_{t}$ is the observation value of the time series at time *t*; B is the backward shift operator; $\phi_{i}$ is the autoregressive coefficient; $\theta_{i}$ is the moving average coefficient; $\varepsilon_{t}$ is the white noise error term; and c is the constant term.

Furthermore, the ARIMA model can be expressed as ARIMA(*p*,*d*,*q*), where *p* is the order of the autoregressive terms, *d* is the order of differencing, and *q* is the order of the moving average terms. In this study, the augmented Dickey–Fuller test and Kwiatkowski–Phillips–Schmidt–Shin test methods were used to test the stationarity of the time series signals [27,28]. In addition, the sum of the Akaike information criterion and the Bayesian information criterion was taken as the information criterion in this study [29,30].

The EMD residual of the stationary interval represents the underlying trend of the rotor current signal before the occurrence of a strong disturbance. In contrast, the residual directly extracted from the jump interval may contain disturbance-induced trend deformation, and is therefore not directly retained as the final trend estimate.

The fitted model predicts a trend sequence with a length equal to that of the jump interval. The observed stationary trend and the predicted jump trend are concatenated to form the complete trend component:

(13) $$T_{complete}(t)=\left\{\begin{array}{l} r_{s}(t),1\leq t\leq t_{b},\\ {\overset{⌢}{r}}_{j}(t),t_{b}\leq t\leq N, \end{array}\right.$$

where $r_{s}(t)$ denotes the stationary trend component and ${\overset{⌢}{r}}_{j}(t)$ denotes the predicted trend component of the jump interval.

Finally, the denoised rotor current signal is reconstructed as follows:

(14) $$\hat{x}(t)=D_{smooth}(t)+T_{complete}(t)$$

where $D_{smooth}(t)$ is the filtered and junction-smoothed dominant component. This reconstruction preserves the full number of observations and their original temporal correspondence.

It should be noted that the ARIMA model was not directly applied to predict the raw data of disturbed jump segments in this study. Instead, the ARIMA model was constructed based on the EMD residual (i.e., trend component) extracted from stationary segments, so as to predict the theoretical trend component that ought to exist within jump segments. The core hypothesis underlying this design is as follows: without external transient disturbances, the trend variation in the rotor current is smooth and predictable; abnormal fluctuations in jump segments are mainly manifested as drastic changes in high-frequency components, while the trend component should remain continuous before and after jumps. This hypothesis conforms to the physical mechanism of north-seeking for magnetically suspended gyros. External disturbances (such as vibration and airflow) primarily affect high-frequency measurement noise rather than the inherent trend drift of the instrument.

To facilitate implementation and improve reproducibility, the parameter settings of the individual modules in the proposed framework described in Section 2.1, Section 2.2, Section 2.3, Section 2.4 and Section 2.5 are summarized in Table 1.

### 2.6. Evaluation Indexes for Reconstruction Effect of Rotor Current Signal

The standard deviation is a key index commonly used in statistics to evaluate the dispersion degree of a dataset s. It effectively quantifies the difference between each value $s_{i}$ in the dataset and the average value $s_{mean}$. When the standard deviation is small, it means that the dispersion degree of the dataset is relatively low, that is, the sampling data are more concentrated. In this experiment, the standard deviation was used as the evaluation criterion of the internal compliance accuracy to compare the effects of different denoising methods.

(15) $$SD=\sqrt{\sum_{i=1}^{n}(s_{i}-s_{mean}{)}^{2}/n-1}$$

The ultimate purpose of denoising the rotor current signal is to improve the azimuth measurement accuracy, so the absolute error is used as the evaluation index of the external compliance accuracy. In this experiment, the instrument erection points were all located in the tunnel control network that was successfully penetrated. The orientation accuracy of the control network was better than 1.2″, which was better than the nominal accuracy of the instrument (5.0″). Therefore, the true azimuth angle of the control network was taken as the true value $\alpha_{0}$ in this experiment. After denoising the rotor current signal, the corresponding azimuth measurement results $\alpha^{*}$ were obtained. Then, the absolute error between the measured value and the true value can be calculated using the following:

(16) $$D=\left|\alpha^{*}-\alpha_{0}\right|$$

The smaller the absolute error, the more significant the denoising effect.

### 2.7. Implementation Procedure

For reproducible implementation, the proposed method can be summarized as Algorithm 1.
**Algorithm 1.** The BEAST-based segmented denoising of maglev gyro rotor current signals.**Input:** Raw rotor current sequence $X=\left\{x_{1},x_{2},\ldots ,x_{N}\right\}$.
Preserve the original time order and check the sequence for invalid or missing records.Apply the BEAST to X and calculate the posterior trend change-point probability at every sampling position.Determine the first persistent statistically supported trend-change position $t_{b}$.Divide X into stationary data $X_{s}$ and jump data $X_{j}$.Apply EMD independently to $X_{s}$ and $X_{j}$.Calculate the Hausdorff-distance similarity sequence for the IMF components of each segment.Remove the identified high-frequency noise-dominant IMFs and reconstruct the dominant component of each segment.Apply optimized wavelet filtering separately to the stationary- and jump-dominant components using segment-specific settings.Concatenate the filtered segments and smooth the local junction region using a moving-average transition window.Use the residual component of $X_{s}$ to fit the ARIMA model.Predict the trend component corresponding to $X_{j}$.Concatenate the observed and predicted trend components.Superimpose the complete trend component and the smoothed dominant component to obtain $\hat{X}$.Calculate the standard deviation and absolute azimuth error of $\hat{X}$.


The proposed method does not require manual deletion, interpolation, normalization, or resampling of the rotor current data before segmentation. The original sampling order and signal length are retained. Any instrument-specific preprocessing, including unit conversion and invalid-record checking, should be completed consistently for all test sequences before the BEAST analysis. Detailed information on the instrument, sampling configuration, observation duration, and field acquisition procedure is provided in Section 3.1.

**Figure 1:**
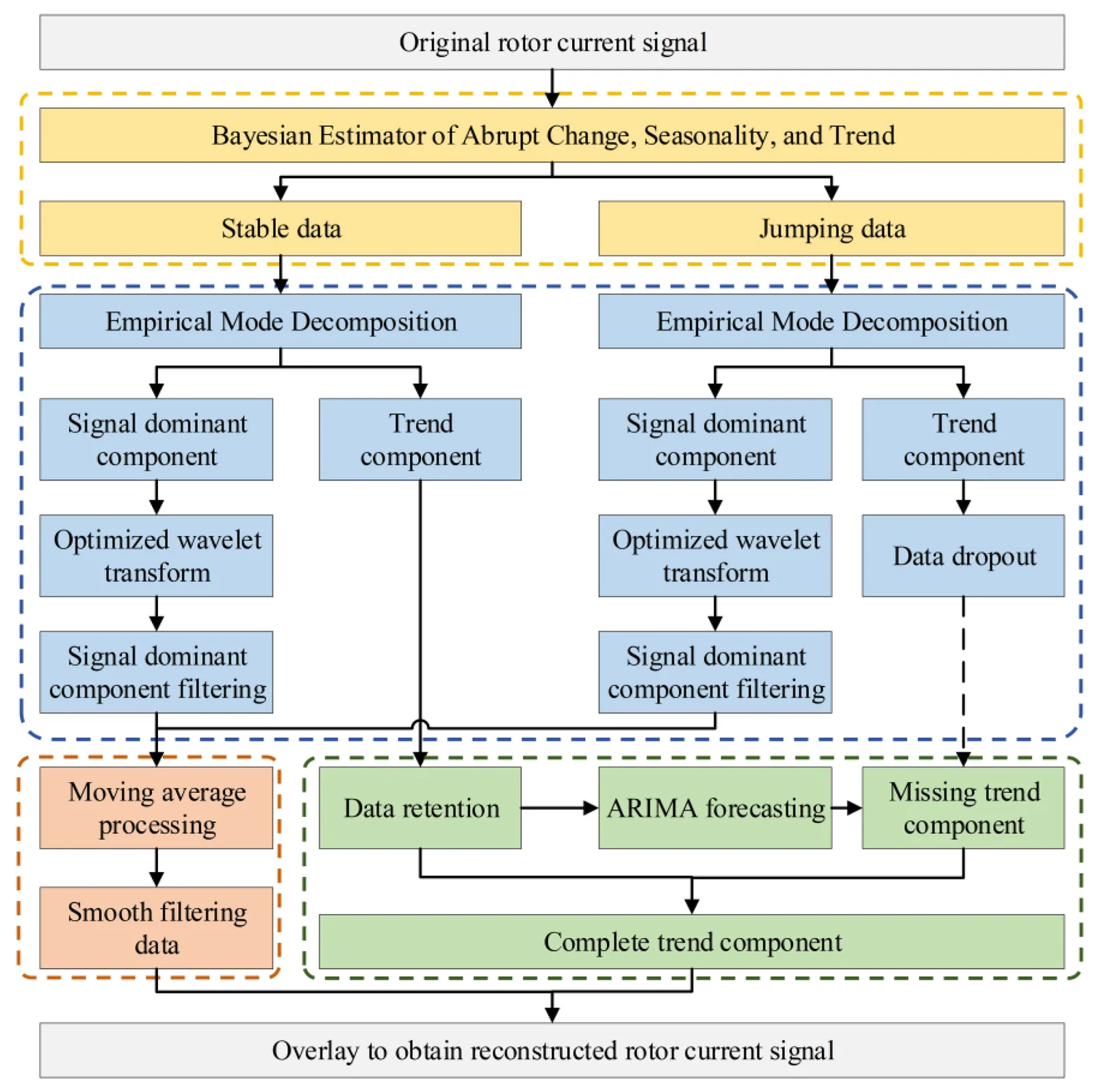
Flowchart of the maglev gyro jump data denoising algorithm.

The technical framework for implementing the proposed maglev gyro jump data denoising algorithm in this study is shown in Figure 1. Figure 1 presents the complete data flow of the proposed method. The yellow module performs probabilistic segmentation, the blue modules implement differentiated component processing, the orange module preserves continuity at the segment junction, and the green modules reconstruct the complete trend component. The segmentation output determines which processing branch is assigned to each sample. The four colored modules correspond to segmentation, adaptive filtering, continuity preservation, and trend reconstruction, respectively.

The proposed method establishes a state-dependent coupling mechanism in which the probabilistic segmentation result directly determines the subsequent decomposition, component screening, denoising, and trend reconstruction procedures. In the original MAF-ARIMA framework, a manually defined window and a uniform filtering configuration are applied to the signal. In contrast, the proposed method uses the BEAST-derived structural boundary as a decision variable that directly determines the decomposition domains, IMF-screening results, wavelet decomposition levels, continuity-processing region, and trend-reconstruction strategy. Therefore, the processing branch and parameter configuration assigned to each observation are determined by its inferred signal state. This “segment first, then filter” mechanism constitutes the principal distinction from the baseline model, rather than the mere addition of BEAST and OWT as independent modules.

## 3. Results and Discussion

### 3.1. Experimental Site, Instrument, and Data Acquisition

Field experiments were conducted at two established control points, denoted as Points A and B, in a railway tunnel. Both points were located within a successfully connected tunnel control network. The orientation accuracy of the reference control network was better than 1.2″, which was higher than the nominal north-seeking accuracy of the maglev gyroscope used in the experiment. Therefore, the known azimuths of the control lines were used as reference values for calculating the absolute azimuth errors.

The instrument used for data acquisition was a D05 maglev gyro total station with a nominal north-seeking accuracy of 5″. During each observation, the instrument was centered and leveled at the control point, initialized according to the manufacturer’s operating procedure, and used to continuously record the torque-rotor current during the north-seeking process. The rotor current was sampled at 167 Hz for approximately 120 s, producing 20,000 observations in each sequence. The current values were stored in dat and expressed in amperes.

External instantaneous disturbances occurred naturally during the tunnel construction and mainly originated from passing vehicles, ventilation airflow, personnel activity, and nearby engineering machinery. These disturbances generated signals with different jump onset times, amplitudes, and durations, thereby providing heterogeneous field samples for evaluating the adaptability of the proposed method.

**Figure 2:**
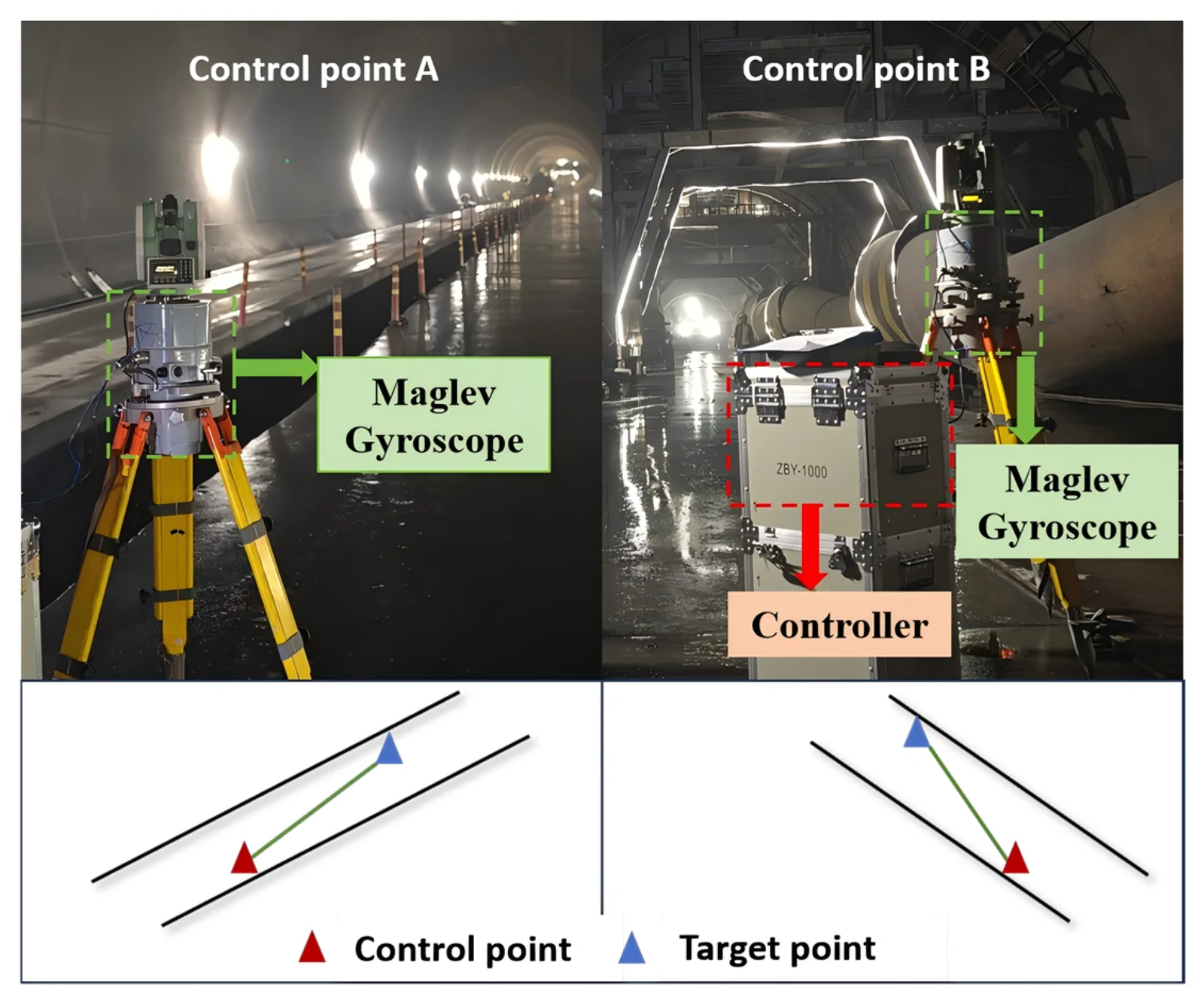
Schematic diagram of instrument erection position.

A total of 12 rotor current sequences containing identifiable abnormal jumps were collected, including six sequences at Point A and six sequences at Point B, as summarized in Table 2. Each sequence corresponded to an independent north-seeking observation. The same raw sequences were processed by the OHHT, HSA-KS, MAF-ARIMA, and the proposed method to ensure a paired and consistent comparison. Figure 2 shows the instrument deployment and control-line configuration at the two observation points.

### 3.2. Data Preprocessing and Parameter Configuration

To preserve the temporal structure of the rotor current observations, no abnormal jump samples were manually removed, interpolated, or replaced before segmentation. The original sampling order and sequence length were retained throughout the entire processing procedure. Prior to algorithm implementation, the raw records were examined for missing values, duplicated timestamps, and invalid numerical entries. No normalization or resampling was applied because the amplitude information of the rotor current was essential for subsequent component decomposition and signal reconstruction.

The original rotor current sequence was directly used as the input to the proposed method. All of the algorithms were implemented in MATLAB R2024b, and identical software and hardware environments were adopted for both the proposed method and all comparison methods to ensure a fair evaluation.

The parameter settings adopted in the proposed method were selected according to the original implementations or well-established empirical configurations reported in previous studies, as summarized in Table 1 (Section 2.5). Therefore, this study focuses on validating the effectiveness of adaptive segmentation and differentiated signal processing rather than performing extensive parameter optimization.

To ensure reproducibility and a fair comparison, the parameter configurations of the benchmark methods followed those reported in their original publications whenever possible. The parameter settings for all benchmark methods were adopted from the corresponding published studies and are summarized in Table 3.

### 3.3. Representative Signal-Processing Results

**Figure 3:**
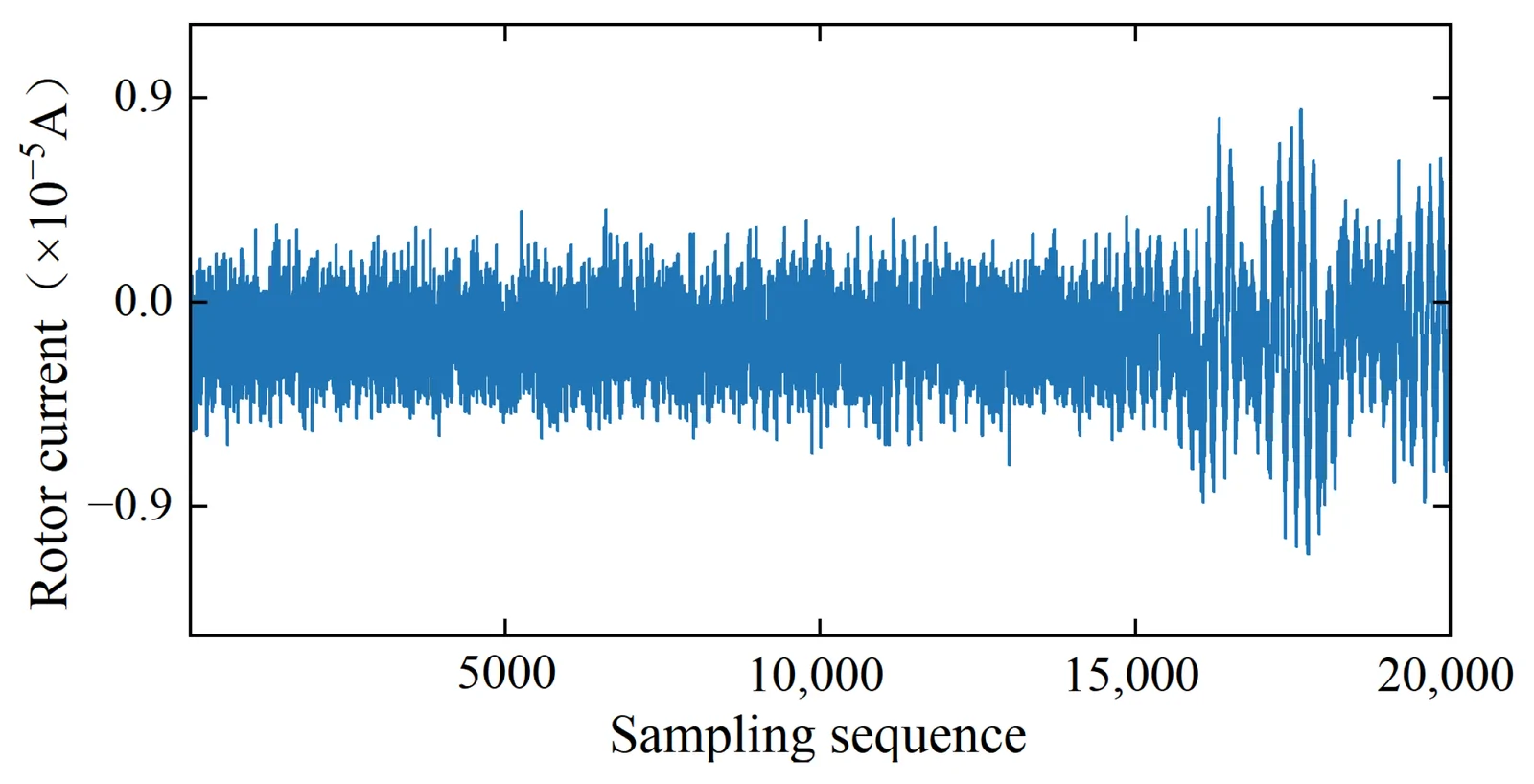
Gyro sampling data.

To illustrate the complete processing procedure, one rotor current sequence collected at Point A was selected as a representative example. The sequence contained 20,000 samples and exhibited relatively stable fluctuations in the early stage, followed by a marked increase in amplitude and dispersion during the later stage, as shown in Figure 3. This example was used only to demonstrate the intermediate outputs of the algorithm; the overall performance assessment was based on all independent test sequences.

**Figure 4:**
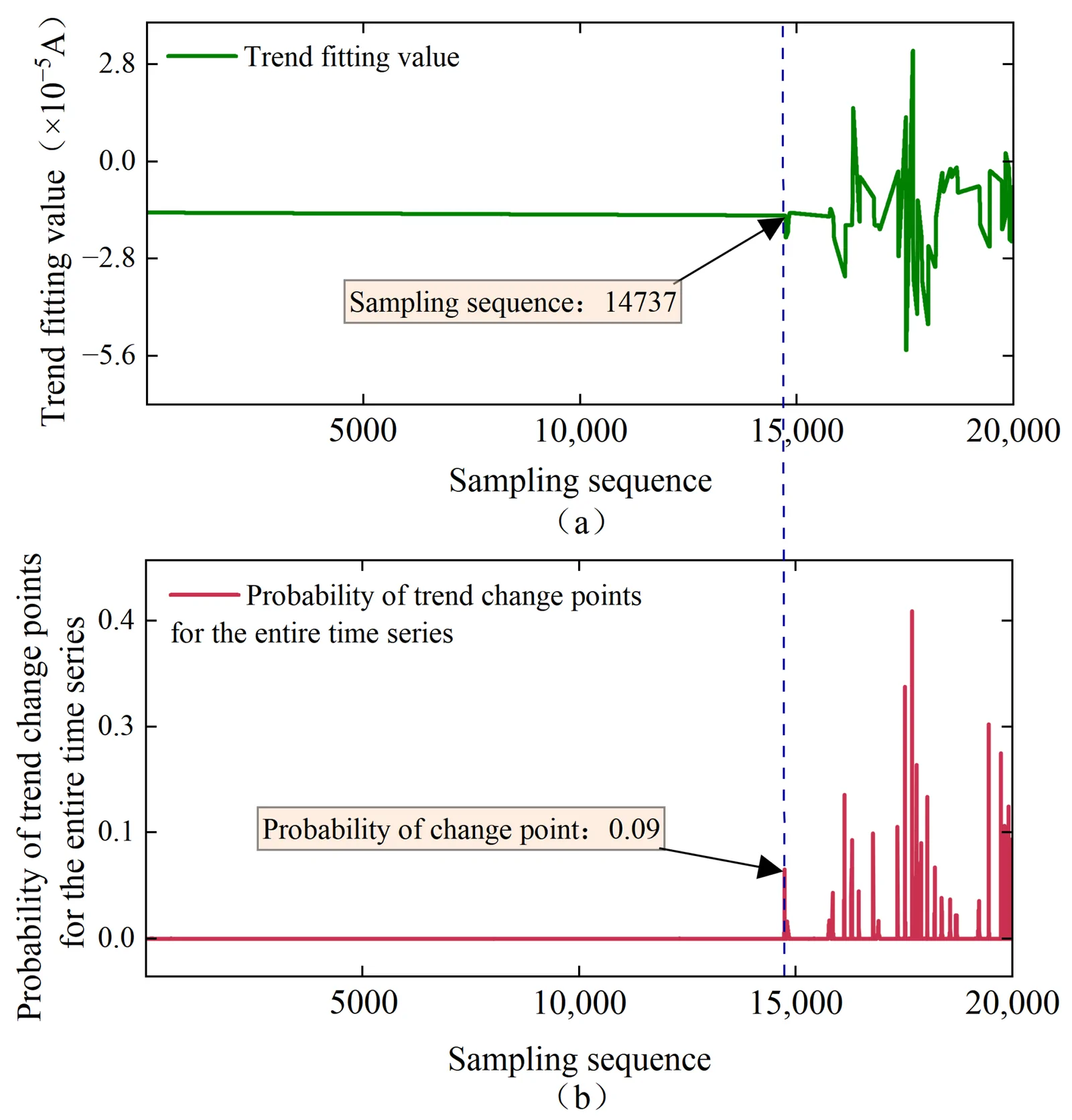
Trend change-point detection of rotor current signal: (**a**) trend-fitting value; (**b**) trend change-point probability of full time series.

To accurately identify the trend mutation in the rotor current signal, this study used the BEAST to analyze the original signal. Figure 4a shows the trend-fitting result output by the BEAST, from which the overall change trend of the signal can be clearly extracted. In the early stage of north-seeking data sampling, the trend-fitting value remained stable; however, at sampling sequence position 14,737, the value changed significantly. Figure 4b shows the posterior change-point probability over the complete rotor current sequence. sample 14,737 is the first candidate that simultaneously exhibits a local posterior-probability peak (9%), a distinct trend change, and persistence over the subsequent observation interval. It is therefore selected as the segmentation boundary.

**Figure 5:**
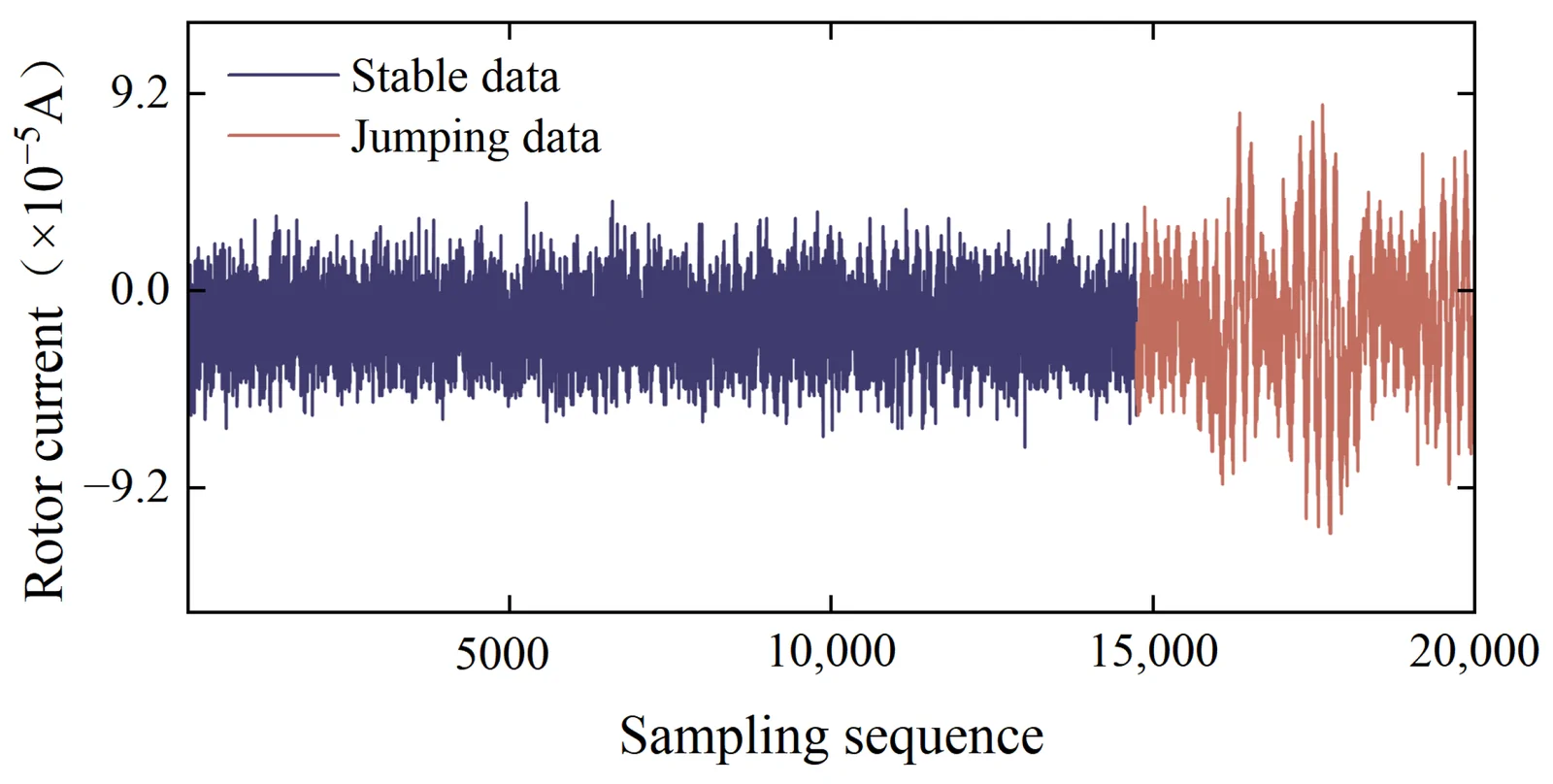
Original signal interval division result.

Combined with the trend analysis results in Figure 4, this study determined sampling sequence position 14,737 as the boundary point between the stationary data and the jump data of the rotor current sampling signal, thus dividing the original rotor current signal into two sections: stationary data (sampling sequences 1–14,736) and jump data (sampling sequences 14,737–20,000). The division result is shown in Figure 5.

The segmentation result provides direct evidence for improved adaptability at the algorithm-design level: the onset of the disturbance is automatically located according to the data themselves, allowing signals with different jump positions to be processed without manually redefining the segmentation window.

**Figure 6:**
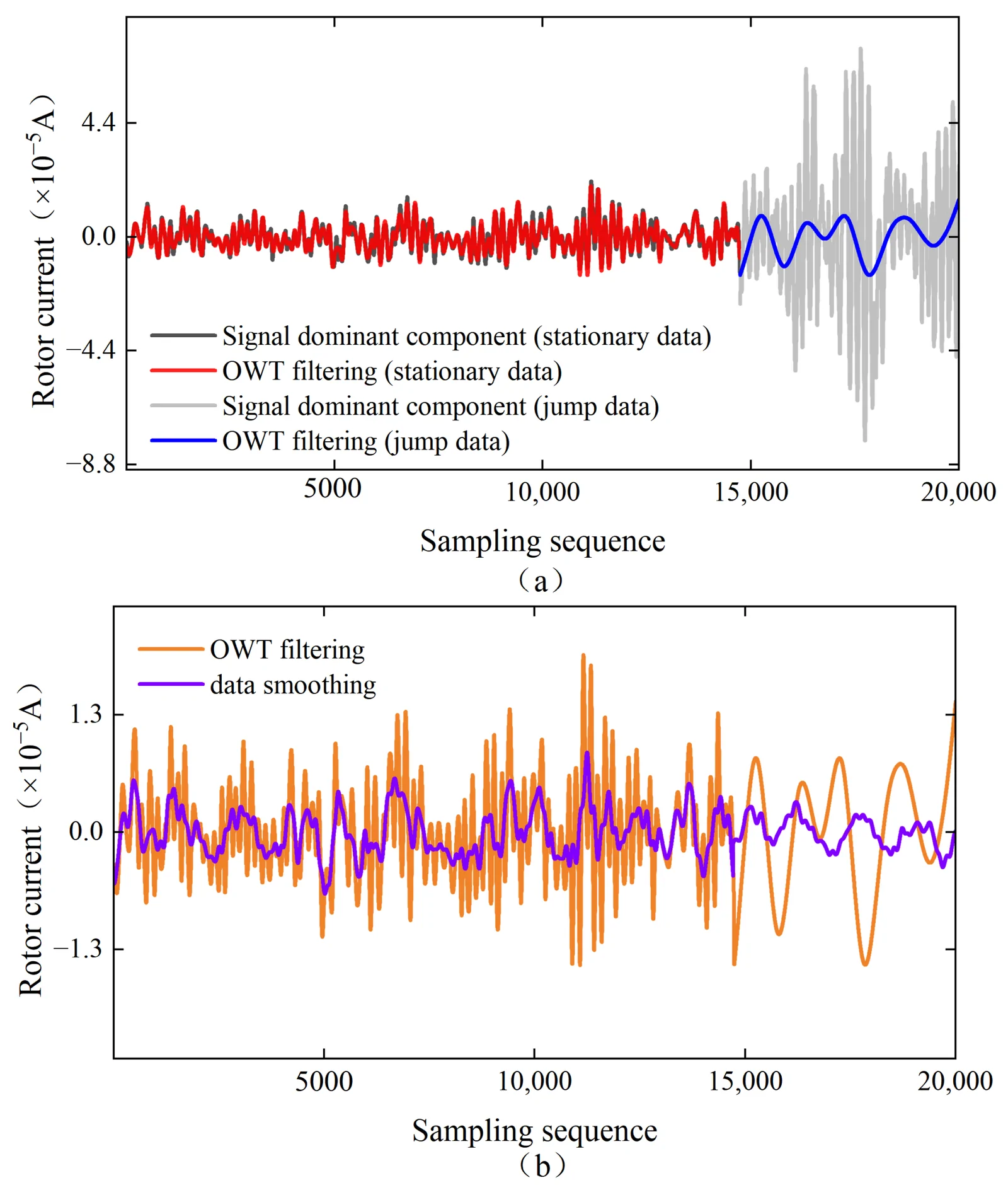
Filtering results of rotor current signal-dominant component and data smoothing: (**a**) rotor current signal-filtering result; (**b**) rotor current signal data smoothing.

The EMD algorithm was used to decompose the stationary data and jump data segments shown in Figure 5. The stationary data segment obtained 10 IMF components, and the jump data segment obtained nine IMF components. According to the Hausdorff-distance calculation results listed in Table 4 and combined with the noise- and signal-dominant component discrimination method defined by Equation (9), the following was clearly identified: for stationary data, IMF1–IMF4 are high-frequency noise-dominant components; for jump data, IMF1–IMF3 are high-frequency noise-dominant components. After eliminating the above high-frequency noise components, the remaining IMF components were superimposed and reconstructed to obtain the signal-dominant component shown in Figure 6a. Furthermore, the OWT method was used to filter the signal-dominant component, and the final filtering result is shown in Figure 6a. Employing varied decomposition levels of an optimal wavelet transform (OWT) effectively alleviates the inherent parameter mismatch problem caused by static predefined parameters in the previous MAF-ARIMA method. Enforcing identical reconstruction orders for the two signal segments brings about two adverse outcomes: the excessive elimination of valuable information from stationary data and insufficient noise suppression for jump-containing signals. In contrast, the proposed method adaptively identifies the retained components according to the local distribution characteristics of each signal fragment.

It can be seen from Figure 6a that there were obvious breakpoints at the junction of segmented data in the filtering result of the signal-dominant component, which was caused by the adoption of differentiated filtering parameters for different data segments. To enhance data continuity, the filtering results were further smoothed. The results show that, after smoothing, the breakpoint phenomenon at the junction of segmented data was significantly improved, as shown in Figure 6b, and the statistical characteristics (such as the standard deviation, mean value, etc.) of the rotor current signal-filtering results over the entire sampling interval showed good uniformity. The burr phenomenon in the filtered and smoothed results was significantly reduced, and the discrete fluctuation in the jump data was also significantly improved.

**Figure 7:**
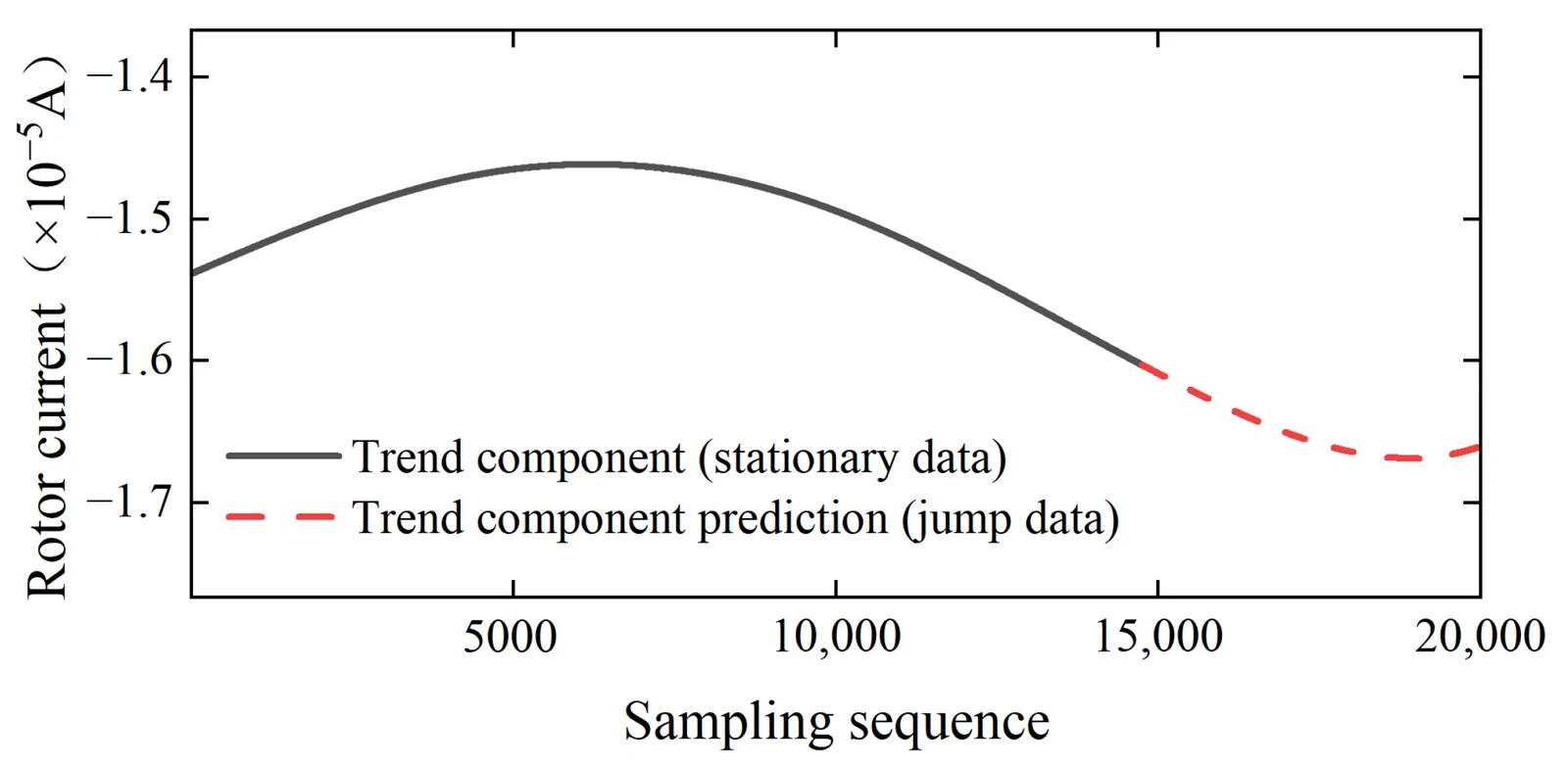
Prediction of missing trend component.

Since a detailed description of the ARIMA model parameter determination process is given in the literature, and this study focused on rotor current signal trend change-point detection and signal-dominant component adaptive filtering processing, the determination process of the ARIMA model parameters is not repeated here. Based on the signal stationarity test results and following the principle of minimizing the information criterion, an ARIMA(0,5,3) prediction model was constructed in this study. The trend component of the stationary data was used as a sample to predict the missing trend component in the jump data segment, and the final prediction result is shown in Figure 7.

The ARIMA model orders were determined following a data-driven procedure. The stationarity of the trend component was first examined using the augmented Dickey–Fuller and Kwiatkowski–Phillips–Schmidt–Shin tests. For the rotor current sequences in this study, the test results indicated that fifth-order differencing was required to achieve stationarity. Although integration orders of *d* = 1 or 2 are more common in typical ARIMA applications, higher-order differencing may be necessary when the time series exhibits strong non-stationarity or complex trend behavior. The autoregressive order *p* = 0 and the moving-average order *q* = 3 were then selected by minimizing the combined Akaike information criterion and Bayesian information criterion (AIC + BIC). This procedure yielded the ARIMA(0,5,3) specification used in this representative signal. All order selections were performed on the stationary-interval trend component and applied consistently to predict the missing trend component of the jump interval. It should be noted that the relatively high differencing order (*d* = 5) in the ARIMA(0,5,3) specification arose from the strong non-stationarity exhibited by the trend component extracted from the stationary interval. The ADF test yielded a *p*-value of 0.482 before differencing, failing to reject the null hypothesis of a unit root, while the KPSS test produced a *p*-value below 0.01, rejecting stationarity. Fifth-order differencing was required to achieve joint stationarity (ADF *p* < 0.01; KPSS *p* > 0.10). Importantly, this ARIMA model was constructed exclusively on the trend component of the stationary segment and was used solely to predict the missing trend values over the jump interval. The raw observations within the stationary interval were neither replaced nor modified by the prediction. Consequently, the risk of over-differencing-induced information loss was confined to the reconstructed trend of the disturbed segment, while the authentic trend characteristics of the undisturbed signal remained fully preserved.

**Figure 8:**
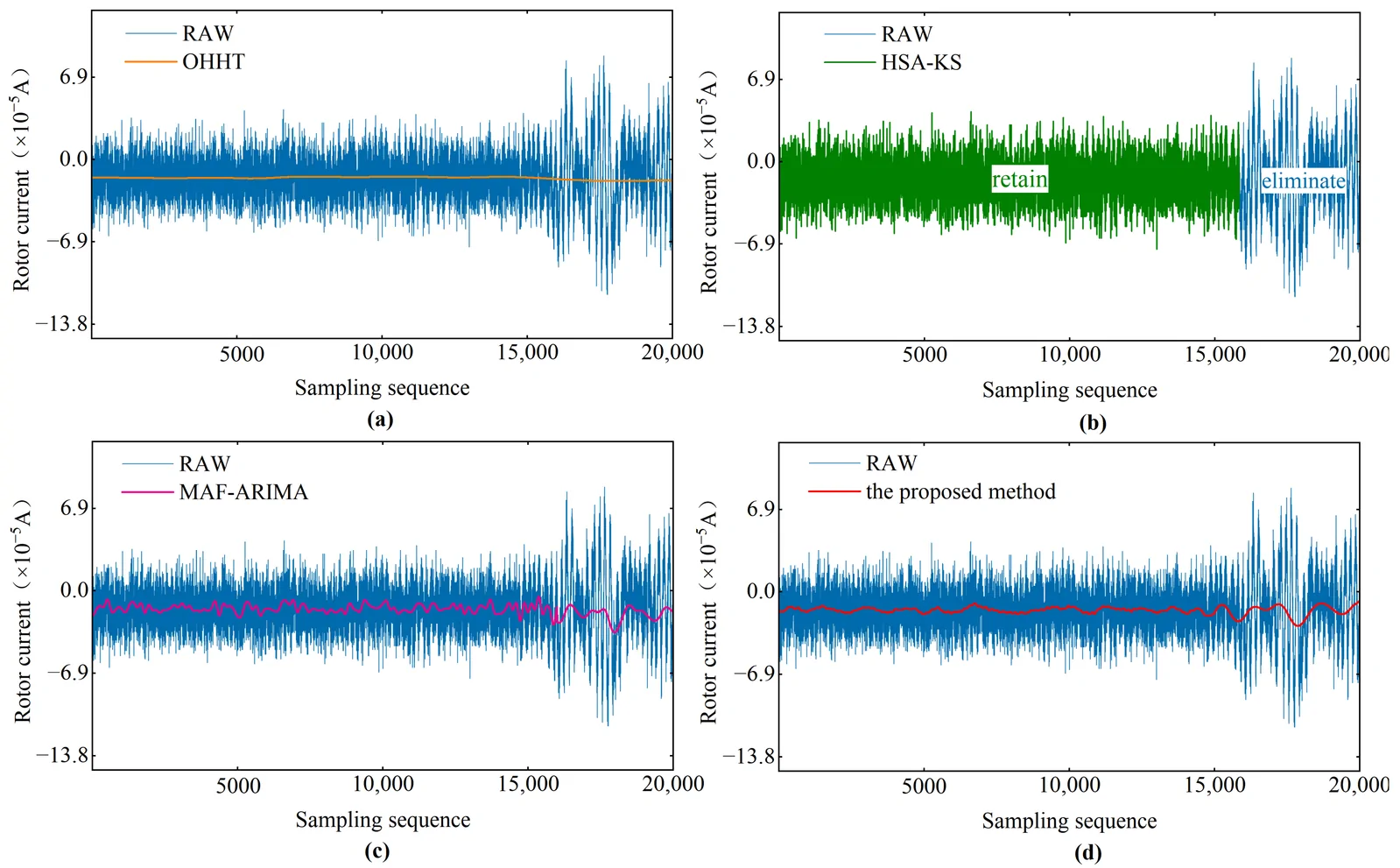
Denoising results of rotor current jump signal: (**a**) OHHT; (**b**) HSA-KS; (**c**) MAF-ARIMA; and (**d**) proposed method.

The filtered and smoothed results of the signal-dominant component shown in Figure 6b were superimposed with the complete trend component shown in Figure 7 to obtain the final denoising result of the rotor current signal, as shown in Figure 8d. Therefore, continuity improvement was achieved not by discarding jump samples, but by combining local junction smoothing with trend reconstruction. This distinction is important because the disturbed interval may still contain valid azimuth-related information.

To comprehensively evaluate the accuracy of the reconstructed signal, the internal and external compliance accuracies were further analyzed, and the results are listed in Table 5 and Table 6, respectively. Compared with other methods, the denoised signal processed by the proposed method shows significant advantages. After processing by the proposed method, the standard deviation of the rotor current reconstructed signal was only 2.89 × 10^−6^ A, indicating that the data fluctuation in the reconstructed signal was effectively suppressed to a low level over the entire sampling interval. In addition, after processing by the proposed method, the absolute error corresponding to the reconstructed signal was significantly reduced from the original 7.03″ to 2.83″, further indicating that the method has a good suppression effect on abnormal data jumps caused by external disturbances.

### 3.4. Comparative Performance on All Test Signals

Although the representative example illustrates the complete processing workflow, the quantitative performance should be evaluated using all independent test sequences.

Table 5 and Table 6 summarize the standard deviations and absolute azimuth errors obtained using the raw signals and the four denoising methods. The standard deviation quantifies the internal dispersion of the reconstructed rotor current signal, whereas the absolute azimuth error evaluates whether denoising ultimately improves the north-seeking result.

According to the current tabulated results, the proposed method reduced the mean standard deviation from 22.52 × 10^−6^ A to 6.54 × 10^−6^ A, corresponding to an average improvement of 70.96%. The mean absolute azimuth error decreased from 8.30″ to 4.12″, corresponding to an average improvement of 50.36%. The proposed method achieved effective azimuth-error reduction for test sequences.

In comparison, MAF-ARIMA reduced the mean standard deviation and mean absolute error by 57.86% and 31.57%, respectively, but failed to achieve effective denoising for one test sequence. HSA-KS achieved improvements of 24.78% and 22.17% and produced negative denoising gains for two sequences. The OHHT reduced the mean standard deviation and absolute error by 38.45% and 16.51%, respectively.

The comparison showed that a low standard deviation alone does not necessarily indicate reliable reconstruction. For example, the OHHT yielded an extremely small standard deviation for some sequences because its high-order reconstruction produced an excessively smooth, approximately linear output. However, the corresponding azimuth-error improvement was limited, indicating that useful signal characteristics may also have been suppressed. Therefore, both internal and external compliance metrics are required for a balanced evaluation.

The sequence-specific negative gains observed for some benchmark methods can be explained by a mismatch between their processing assumptions and the disturbance characteristics. HSA-KS removes samples identified as abnormal. When the detected interval is long or contains valid mean-current information, sample deletion changes the temporal weighting of the sequence and may bias the mean rotor current used for azimuth calculation. MAF-ARIMA preserves the sequence length, but its fixed segmentation window and uniform filtering configuration may either include disturbed observations in the stationary reference interval or impose excessive smoothing on undisturbed data. Under such conditions, the reconstructed trend can deviate from the expected continuation, leading to limited or negative azimuth-error improvement. These observations demonstrate that a reduction in standard deviation alone does not necessarily indicate an improvement in north-seeking accuracy.

To verify the statistical robustness of the aforementioned performance improvements and control the risk of false positives under multiple comparisons, this study further included the conduction of nonparametric significance tests on the paired repeated-measurement data of error metrics. Specifically, the Friedman test was adopted for overall inter-group comparisons, and the Wilcoxon signed-rank test corrected via the Holm method was utilized for pairwise comparisons. Table 7 and Table 8 summarize the descriptive statistics of the standard deviation and absolute error metrics along with the overall Friedman test results for each algorithm, respectively. Table 9 further presents the significance conclusions of the post hoc paired comparisons between the BEAST and all comparative algorithms after a Holm correction.

For the standard deviations, the Friedman test revealed a significant overall difference among the OHHT, HSA-KS, MAF-ARIMA, and proposed method (χ^2^(3) = 19.60, *p* < 0.001). The proposed method yielded the lowest mean value (6.54 ± 3.75), compared with 13.86 ± 10.57 for the OHHT, 16.94 ± 4.24 for HSA-KS, and 9.49 ± 5.91 for MAF-ARIMA. Subsequent Wilcoxon signed-rank tests with a Holm correction showed that the proposed method produced significantly lower values than the OHHT, HSA-KS, and MAF-ARIMA (all adjusted *p* < 0.05). Although the proposed method was not the best-performing method for every individual signal group, its overall paired performance was significantly better across the 12 groups.

For the absolute-error results, ten signal groups had complete observations for all denoising methods. The Friedman test again indicated a significant overall difference (χ^2^(3) = 23.88, *p* < 0.001). The BEAST achieved the lowest mean absolute error (4.12 ± 1.37), followed by the MAF-ARIMA (5.68 ± 1.30), HSA-KS (6.46 ± 1.77), and the OHHT (6.93 ± 2.77). Pairwise Wilcoxon signed-rank tests demonstrated that the absolute errors obtained with the proposed method were significantly lower than those obtained with each competing method after a Holm correction (all adjusted *p* = 0.002). These findings confirm that the improvement achieved by the proposed method was systematic and statistically significant rather than being driven by only a small number of signal groups.

### 3.5. Ablation Study

The proposed method in this study is developed based on the EMD-MAF-ARIMA framework. Therefore, hierarchical ablation experiments are designed to quantitatively evaluate the contribution of each individual module to the overall model performance. The detailed configurations of each ablation scheme are described as follows:

- Original data scheme.
- Baseline model (EMD + MAF + ARIMA).
- Baseline model integrated with the BEAST module (BEAST + EMD + MAF + ARIMA).
- Baseline model integrated with the OWT module (EMD + OWT + -MAF + ARIMA).
- The complete proposed method integrating both BEAST and OWT modules.

The quantitative results of the ablation experiments are summarized in Table 10 and Table 11.

The experimental results demonstrate that each introduced module contributes positively to the overall performance improvement of the proposed method. Compared with the original data scheme, the baseline model reduces the Error Mean, Std Mean, and Jump Mean by 31.57%, 57.86%, and 94.34%, respectively. This result verifies the effectiveness of the EMD-MAF-ARIMA framework in extracting informative signal features and suppressing noise interference.

By further incorporating the BEAST module into the baseline model, the three evaluation metrics are additionally reduced by 17.43%, 16.75%, and 21.79%, respectively. This improvement indicates that the BEAST module enhances the capability of capturing local transient variations and effectively suppresses abrupt fluctuations in rotor current signals.

Similarly, the introduction of the OWT module also improves the model performance, reducing the Error Mean, Std Mean, and Jump Mean by 11.62%, 9.48%, and 15.75%, respectively. These results indicate that the OWT module contributes to improving temporal consistency and alleviating signal instability during dynamic variation processes.

Finally, the complete proposed method integrating both BEAST and OWT modules achieves the best performance among all ablation schemes. Compared with the baseline model, the three evaluation metrics are reduced by 27.46%, 31.09%, and 32.92%, respectively. These results confirm that BEAST and OWT provide complementary advantages: BEAST mainly improves the localization of disturbance-induced structural changes and enables state-dependent processing, while OWT improves local denoising and feature preservation, whereas junction smoothing directly reduces artificial discontinuity at the segment boundary. The combination of these two modules enables simultaneous improvements in prediction accuracy and operational stability.

The experimental results can be further interpreted in relation to the three limitations identified in the Introduction. First, the BEAST-based segmentation successfully located the onset of disturbance in all 12 sequences without manual parameter adjustment, directly supporting the claim of improved adaptability. Second, the fact that the proposed method achieved effective denoising for every test sequence—whereas MAF-ARIMA exhibited inferior denoising performance in one case—indicates that segment-dependent filtering alleviates the fixed-parameter mismatch problem that arises when identical settings are imposed on intervals with different disturbance intensities. Third, the absolute azimuth error was reduced without discarding any jump samples, confirming that the combination of junction smoothing and trend prediction preserves signal continuity while suppressing noise. Thus, the improvements in standard deviation and azimuth error are not merely numerical gains but evidence that the three targeted limitations have been substantively mitigated.

A closer examination of the ablation results reveals an important insight regarding the relative contributions of the two key modules. Integrating the BEAST module into the baseline model (Scheme C) reduced the mean absolute azimuth error by 17.43%, whereas incorporating the OWT module alone (Scheme D) achieved a reduction of 11.62%. This discrepancy indicates that adaptive time-series segmentation contributes more substantially to the final azimuth accuracy than localized wavelet filtering optimization under the present disturbance conditions. This finding is interpretable from a signal-processing perspective: the primary factor degrading the north-seeking result is not the presence of high-frequency noise per se, but the structural mismatch caused by applying a unified denoising strategy to two fundamentally different signal regimes. Once the stationary and jump intervals are correctly separated by BEAST, even relatively simple filtering applied separately to each segment yields considerable improvement. The OWT module, while valuable for refining local denoising performance, plays a secondary role compared with the segmentation step. This observation strongly supports the core philosophy of the proposed method—“segment first, then filter”—and confirms that accurately localizing the disturbance-induced structural change is the most critical prerequisite for effective rotor current signal reconstruction.

## 4. Conclusions

This study proposed a BEAST-based segmented denoising framework for maglev gyro rotor current signals affected by instantaneous disturbances in tunnel environments. The central methodological contribution is a data-driven, state-dependent processing strategy in which the detected disturbance boundary directly determines the subsequent decomposition, component screening, filtering, continuity correction, and trend reconstruction procedures. The main conclusions are as follows.

(1) BEAST enables adaptive localization of disturbance onset without manually defined segmentation windows. The rotor current sequence is divided into stationary and disturbed intervals according to posterior evidence of a persistent structural trend change. In the representative sequence, the disturbance boundary was identified at sample 14,737, while all 20,000 observations and their original temporal order were retained.
(2) Segment-wise processing reduces the parameter mismatch inherent in uniform denoising. Independent EMD and Hausdorff-distance-based IMF screening produced different component-removal orders for the stationary and jump intervals. Applying segment-specific wavelet decomposition levels therefore avoided excessive smoothing of the stationary interval and insufficient noise suppression in the strongly disturbed interval.
(3) The synergistic integration of global smoothing and trend forecasting ensures the continuity and integrity of the signal. Moving-average processing corrected the artificial discontinuity introduced at the segment junction, whereas ARIMA reconstructed the trend component of the disturbed interval from the stationary reference trend. Unlike sample-deletion methods, the proposed approach retained the complete sequence and preserved potentially useful azimuth-related information.
(4) The proposed framework achieved consistent improvements in both signal stability and north-seeking accuracy. Across 12 field-collected sequences, the mean standard deviation decreased from 22.52 × 10^−6^ A to 6.54 × 10^−6^ A, corresponding to a reduction of 70.96%. The mean absolute azimuth error decreased from 8.30″ to 4.12″, representing an improvement of 50.36%. Valid azimuth results and error reductions were obtained for all 12 sequences, and the proposed method significantly outperformed OHHT, HSA-KS, and MAF-ARIMA in the paired statistical comparisons.

Overall, the contribution of the proposed framework is not the simple addition of BEAST and OWT to MAF-ARIMA. Its novelty lies in coupling probabilistic change-point inference with segment-dependent IMF screening, differentiated wavelet filtering, local continuity correction, and trend reconstruction. The results support the core design principle of the framework: “segment first, then filter.”

Although the proposed framework demonstrated consistent improvements, several limitations warrant further investigation. First, the current framework adopts a binary stationary/disturbed segmentation strategy. Future studies will extend it to multiple-change-point and multi-state processing to characterize signals containing repeated or alternating disturbances. Second, ARIMA provides an efficient and interpretable predictor for the present dataset, but its linear structure may limit its ability to represent more complex trends. Nonlinear models, including LSTM- and Transformer-based predictors, will be evaluated when larger training datasets become available. Third, the generalizability of the framework will be assessed using data from additional tunnels, instruments, sampling configurations, sensors, and disturbance conditions.

## Figures and Tables

**Table 1:** Parameter configuration of the proposed method.

Module	Parameter	Value/Rationale
Implementation	Software environment	MATLAB R2024b with the Rbeast toolbox
BEAST	Seasonal component	Disabled (no significant periodicity observed in the rotor current)
	Truncated Poisson prior	λ = 0.5 × N × 10^−4^ (N = 20,000)
	MCMC iterations/Burn-in	10,000/2000
	Remaining Bayesian priors	Default configuration of the official Rbeast implementation
EMD	Decomposition stopping criterion	Residual becomes monotonic or contains no more than one extremum
Hausdorff-based IMF selection	IMF identification criterion	PDF similarity based on the Hausdorff distance (Equation (9))
OWT (Stationary interval)	Wavelet basis/Decomposition level	sym10/6
OWT (Jump interval)	Wavelet basis/Decomposition level	sym10/9
OWT (Common settings)	Threshold selection/Thresholding	rigrsure/Soft threshold
Moving-average smoothing	Window length	500 samples
ARIMA	Model order selection	Determined using the ADF and KPSS stationarity tests together with the AIC–BIC criterion

**Table 2:** Experimental instrument and data-acquisition configuration.

Item	Configuration
Test environment	Railway tunnel
Observation sites	Control Points A and B
Instrument model	GAT D05
Nominal north-seeking accuracy	5.0″
Sampling frequency	167 Hz
Sampling duration	120 s
Samples per sequence	20,000
Number of sequences	12
Rotor current unit	A
Reference azimuth accuracy	Better than 1.2″
Main disturbance sources	Vehicles, airflow, personnel, and engineering machinery

**Table 3:** Parameter configuration of the benchmark methods.

Method	Parameter	Value/Rationale
OHHT	EMD reconstruction order	8
	Wavelet basis	db4
	Wavelet decomposition level	4
	Threshold selection	heursure
HSA-KS	Segmentation significance level	0.05
	Kolmogorov–Smirnov confidence level	95%
	Minimum segmentation window	100 samples
MAF-ARIMA	Moving-average window length	500 samples
	ARIMA model order	Determined using the AIC–BIC criterion

**Table 4:** Hausdorff distances corresponding to IMF components obtained by partition decomposition of rotor current signal.

**IMF Component**	**1**	**2**	**3**	**4**	**5**
HD1 (×10^3^)	7.88	44.46	79.08	113.43	84.25
HD2 (×10^3^)	8.17	45.02	81.54	50.98	0.64
**IMF Component**	**6**	**7**	**8**	**9**	**10**
HD1 (×10^3^)	120.40	190.45	356.41	1349.62	697.92
HD2 (×10^3^)	75.23	17.26	78.08	348.55	/—

“—”indicates that no data are available for the corresponding entry.

**Table 5:** Standard deviations of denoised signals corresponding to different denoising algorithms (×10^−6^ A).

Signal Group	RAW	OHHT	HSA-KS	MAF-ARIMA	Proposed Method
1	19.72	1.13	14.71	4.29	2.89
2	13.48	1.86	12.35	2.83	4.26
3	17.03	11.61	14.74	9.70	4.28
4	18.52	16.34	12.55	7.83	4.10
5	24.34	22.59	16.38	10.07	5.07
6	15.91	3.77	15.30	3.21	3.44
7	16.37	5.79	16.22	5.82	6.27
8	35.51	34.30	21.95	21.01	13.21
9	25.76	11.84	17.86	9.57	7.36
10	38.54	29.34	27.34	20.55	14.57
11	24.75	15.69	19.13	10.51	7.80
12	20.34	12.11	14.72	8.54	5.23
Mean	22.52	13.86	16.94	9.49	6.54
Improvement (%)	—	38.45	24.78	57.86	70.96

**Table 6:** Absolute errors of denoised signals corresponding to different denoising algorithms (″).

Signal Group	RAW	OHHT	HSA-KS	MAF-ARIMA	Proposed Method
1	7.03	3.74	5.26	4.60	2.83
2	3.49	3.47	/	/	2.50
3	13.81	13.46	5.73	5.56	5.43
4	3.60	3.56	2.75	2.94	1.67
5	7.85	7.15	6.74	6.88	4.17
6	10.11	6.59	9.09	5.72	3.93
7	8.47	7.64	/	5.68	4.17
8	7.44	7.21	6.97	5.86	5.40
9	7.42	6.92	6.57	5.58	4.71
10	13.65	9.56	8.53	8.26	6.34
11	9.65	7.62	7.15	5.91	5.11
12	7.03	6.29	5.76	5.48	3.23
Mean	8.30	6.93	6.46	5.68	4.12
Valid n	—	12	10	11	12
Improvement (%)	—	16.51	22.17	31.57	50.36

“/” indicates cases where the processed error is larger than the original error, resulting in a negative gain.

**Table 7:** Descriptive statistics for the standard deviations *SD* (×10^−6^ A).

Method	n	Mean ± SD	Median (IQR)
RAW	12	22.52 ± 7.78	20.03 (8.14)
OHHT	12	13.86 ± 10.57	11.98 (12.62)
HSA-KS	12	16.94 ± 4.24	15.76 (3.47)
MAF-ARIMA	12	9.49 ± 5.91	9.06 (4.74)
Proposed method	12	6.54 ± 3.75	5.15 (3.25)

IQR, interquartile range. Lower values indicate better performance. The four denoising methods differed significantly according to the Friedman test (χ^2^(3) = 19.60, *p* < 0.001).

**Table 8:** Descriptive statistics for the absolute errors *D* (″).

Method	Valid n	Mean ± SD	Median (IQR)
RAW	12	8.30 ± 3.22	7.65 (2.74)
OHHT	12	6.93 ± 2.77	7.04 (1.97)
HSA-KS	10	6.46 ± 1.77	6.66 (1.37)
MAF-ARIMA	11	5.68 ± 1.30	5.68 (0.37)
Proposed method	12	4.12 ± 1.37	4.17 (2.05)

The HSA-KS method had invalid results for signal groups 2 and 7, and the MAF-ARIMA had an invalid result for signal group 2. The Friedman test was therefore performed using the ten signal groups with complete observations. The four denoising methods differed significantly (χ^2^(3) = 23.88, *p* < 0.001).

**Table 9:** Pairwise Wilcoxon signed-rank comparisons between the proposed method and the raw or denoised results.

Outcome	Comparison	Paired n	Raw *p*	Holm-Adjusted *p*
*SD*	Proposed method vs. RAW	12	<0.001	0.002
*SD*	Proposed method vs. OHHT	12	0.016	0.019
*SD*	Proposed method vs. HSA-KS	12	<0.001	0.002
*SD*	Proposed method vs. MAF-ARIMA	12	0.009	0.019
*D*	Proposed method vs. RAW	12	<0.001	0.002
*D*	Proposed method vs. OHHT	12	<0.001	0.002
*D*	Proposed method vs. HSA-KS	10	0.002	0.002
*D*	Proposed method vs. MAF-ARIMA	11	<0.001	0.002

Two-sided Wilcoxon signed-rank tests were used. Holm-adjusted *p*-values below 0.05 were considered statistically significant. Rounded, adjusted *p*-values are reported.

**Table 10:** Overall Performance Comparison of Different Ablation Schemes Based on Averaged Evaluation Metrics.

Scheme	Error Mean (″)	Error Improv. (%)	Std Mean (×10^−6^ A)	Std Improv. (%)	Jump Mean (×10^−6^ A)	Jump Improv. (%)
A	8.30	—	22.52	—	18.80	—
B	5.68	31.57	9.49	57.86	1.06	94.34
C	4.69	43.49	7.90	65.04	0.83	95.58
D	5.02	39.52	8.59	62.00	0.89	95.24
E	4.12	50.36	6.54	70.96	0.71	96.22

Jump Mean denotes the average of the absolute jump amplitudes at multiple breakpoints.

**Table 11:** Detailed Ablation Experimental Results Under Different Test Conditions.

Scheme	Group	Error (″)	Std (×10^−6^ A)	Breakpoint Jump (A)	Group	Error (″)	Std (×10^−6^ A)	Breakpoint Jump (A)
A	G1	7.03	19.72	1.70 × 10^−5^	G2	3.49	13.48	8.00 × 10^−6^
B		4.60	4.29	2.88 × 10^−8^		/	2.83	9.58 × 10^−8^
C		3.17	3.63	1.21 × 10^−8^		2.92	4.34	3.73 × 10^−8^
D		3.85	3.91	2.05 × 10^−8^		2.97	4.01	6.12 × 10^−8^
E		2.83	2.89	2.03 × 10^−9^		2.50	4.26	5.47 × 10^−9^
A	G3	13.81	17.03	3.00 × 10^−6^	G4	3.6	18.52	−3.00 × 10^−6^
B		5.56	9.70	5.04 × 10^−8^		2.94	7.83	−2.54 × 10^−8^
C		5.48	4.41	1.83 × 10^−8^		2.53	4.27	−1.10 × 10^−8^
D		5.52	6.15	3.11 × 10^−10^		2.78	5.92	−1.86 × 10^−8^
E		5.43	4.28	6.85 × 10^−6^		1.67	4.10	−1.76 × 10^−9^
A	G5	7.85	24.34	1.00 × 10^−6^	G6	10.11	15.91	−3.10 × 10^−5^
B		6.88	10.07	7.92 × 10^−8^		5.72	3.21	−1.15 × 10^−5^
C		4.93	5.23	9.39 × 10^−8^		4.54	3.52	−9.06 × 10^−6^
D		5.65	7.14	5.08 × 10^−8^		5.16	4.05	−9.82 × 10^−6^
E		4.17	5.07	3.34 × 10^−8^		3.93	3.44	−8.31 × 10^−6^
A	G7	8.47	16.37	−2.80 × 10^−5^	G8	7.44	35.51	−5.70 × 10^−5^
B		5.68	5.82	−9.11 × 10^−8^		5.86	21.01	−2.62 × 10^−7^
C		5.11	6.99	−7.43 × 10^−8^		5.55	19.77	−1.71 × 10^−7^
D		5.32	6.35	−6.47 × 10^−8^		5.61	20.40	−2.05 × 10^−7^
E		4.17	6.27	−1.80 × 10^−8^		5.4	13.29	−3.43 × 10^−8^
A	G9	7.42	25.76	−1.60 × 10^−5^	G10	13.65	38.54	−2.03 × 10^−6^
B		5.58	9.57	−4.62 × 10^−7^		8.26	20.55	−4.39 × 10^−8^
C		5.09	8.84	−3.18 × 10^−7^		6.98	18.48	−1.87 × 10^−8^
D		5.31	9.12	−3.47 × 10^−7^		7.45	19.23	−2.64 × 10^−8^
E		4.71	7.36	−9.71 × 10^−8^		6.34	14.62	−4.89 × 10^−9^
A	G11	9.65	24.75	4.40 × 10^−5^	G12	7.03	20.34	1.70 × 10^−5^
B		5.91	10.51	6.27 × 10^−8^		5.48	8.54	4.12 × 10^−8^
C		5.37	8.42	7.16 × 10^−8^		4.62	7.05	6.74 × 10^−8^
D		5.64	9.26	4.05 × 10^−8^		4.96	7.61	2.93 × 10^−8^
E		5.11	7.80	−9.87 × 10^−9^		3.23	5.23	1.36 × 10^−8^

“/” indicates cases where the processed error is larger than the original error, resulting in a negative gain.

## Data Availability

The raw data supporting the conclusions of this article will be made available by the authors on request.
